# Importation of Adulterated Drugs and Medicines

**Published:** 1848

**Authors:** 


### ARTICLE III.

### IMPORTATION OF ADULTERATED DRUGS AND MEDICINES.

This subject, which our readers are aware has been attracting a great deal of attention for some time, is brought before Congress. Dr. T. O. Edwards of Ohio, chairman of a select committee on the subject, has made an able and full report setting forth the extensive frauds, practised in vending many of the most important articles of the materia medica.

A bill for its prevention has been introduced in the House of Representatives accompanying this report, which we hope will pass in such form as to put a stop to these frauds, from which incalcuable injuries must result to the health and lives of our citizens. We had intended giving an extended notice of the report, but as most of our readers will receive the document from Washington, and our space is limited, we let this suffice. [E.

